# Dehydrodiisoeugenol targets NOD2 exerting dual effects against colitis and colorectal cancer: a double-edged sword

**DOI:** 10.1186/s10020-025-01193-7

**Published:** 2025-06-05

**Authors:** Feiyang Yi, Nianzhi Chen, Maoyuan Zhao, Zhili Gu, Yun Yuan, Xuegui Tang, Fang Liu

**Affiliations:** 1 Clinical Medicine College of Integrated Chinese and Western Medicine, North Sichuan Medical College, Nanchong, China; 2 Shuguang Hospital Affiliated to Shanghai University of Traditional Chinese Medicine, Shanghai, China; 3 School of Chinese Materia Medica, Beijing University of Chinese Medicine, Beijing, China; 4 Hospital of Chengdu University of Traditional Chinese Medicine, Chengdu, China

**Keywords:** Ulcerative colitis, Colorectal cancer, Dehydrodiisoeugenol, NOD2

## Abstract

Dehydrodiisoeugenol (DEH) is a primary benzofuran-type neolignan isolated from the Chinese herbal medicine *nutmeg*, which is used in the treatment of gastrointestinal diseases. This study aims to observe the dual therapeutic effects of DEH on DSS-induced ulcerative colitis (UC) and colorectal cancer (CRC), with a focus on exploring the molecular mechanism by which DEH exerts anti-inflammatory effect in inflammatory cells and induces autophagy in CRC cells. An inflammatory cell model was established by LPS/IFNγ-stimulated RAW264.7 macrophages to observe the anti-inflammatory effect of DEH, while colon cancer cell lines were used to observe the anticancer activity of DEH. DSS-induced mice and subcutaneous tumor model in nude mice were also established to observe the bidirectional regulation of DEH. This study demonstrates that low concentrations of DEH exhibit anti-inflammatory effects in *vitro*. Importantly, DEH achieves significant inhibition of IκBα degradation and phosphorylation levels, as well as subsequent NF-κB nuclear translocation, while also suppressing the phosphorylation of the MAPK family members JNK, ERK, and p38, by reducing elevated NOD2 expression induced by LPS/IFNγ. In addition, oral administration of DEH improves colitis and colonic barrier damage in DSS-induced mice. Interestingly, at a high concentration of DEH significantly activates the NOD2 signaling pathway to promote autophagy and apoptosis in CRC cells, contributing to its anti-CRC effect. These findings suggest that different concentration of DEH shows bidirectional regulation by improve inflammatory responses in UC and simultaneously possess anti-CRC effects by targeting the NOD2. This highlights DEH as a promising candidate for clinical treatment of UC and CRC.

**Supplementary Information:**

The online version contains supplementary material available at 10.1186/s10020-025-01193-7.

## Introduction

Colorectal cancer (CRC) is a common malignant tumor in the gastrointestinal tract and ranks as the second leading cause of cancer-related deaths (Dekker et al. 2019). The 5-year and 10-year survival rates for CRC patients are 65% and 58%, respectively. In recent years, the overall incidence of CRC has declined among individuals over 50 years of age but has increased among those under 50(Siegel et al. 2017). Due to environmental changes and economic development, it is projected that the number of CRC patients will increase by 60% by the year 2030, becoming a significant public health concern. Therefore, developing strategies for prevention and treatment of CRC is crucial for reducing the disease burden.

The gut is a unique environment in host defense, where inflammation plays a central role in resolving pathogenic infections, maintaining normal intestinal function, or promoting tumor formation (Mantovani et al. 2008; Netea et al. 2017). Inflammatory bowel disease (IBD), as an immune-mediated chronic intestinal inflammation, is a significant factor in the onset of CRC (Si et al. 2022). IBD mainly includes Crohn’s disease (CD) and ulcerative colitis (UC), which involve lesions in the mucosal and submucosal layers of the colon and rectum. The increased risk of CRC in IBD patients confirms the role of inflammation in CRC development(Baker et al. 2019; Rubin et al. 2012). In 2023, the global prevalence of IBD is estimated to be 5 million cases, with an increasing incidence trend (Le Berre et al. 2023). The time it takes for chronic inflammation in the intestines to progress to cancer is not a precise number. It may take anywhere from 10 to 30 years or even longer for chronic inflammation to lead to cancer. Research indicates that up to 42% of IBD patients are found to have high-grade dysplasia, either already suffering from CRC or progressing to CRC within a short period. Therefore, controlling the chronic persistent inflammation of IBD and inhibiting the “inflammation-carcinogenesis” process are crucial steps in preventing CRC. Finding potential, highly effective, and low adverse reaction anti-inflammatory drugs is of utmost importance for preventing CRC, considering the limitations of current therapeutic interventions. There are many common signaling pathways between IBD and CRC, including cell survival, cell proliferation, angiogenesis, and inflammatory signaling pathways. Therefore, identifying common biomarkers for both IBD and CRC is crucial.

Nucleotide-binding oligomerization domain protein 2 (NOD2), also known as CARD15, is primarily expressed in monocyte macrophages, intestinal mucosal epithelium, paneth cells, and dendritic cells. NOD2, as an important gene involved in regulating inflammation and autophagy(Fritz et al. 2011; Mukherjee et al. 2019; Negroni et al. 2018), was the first susceptibility gene discovered to be associated with IBD (Branquinho et al. 2016; Goethel et al. 2019; Venkataraman and Rivas 2019). A number of research found that NOD2-mediated nuclear autophagy-induced DNA damage promotes the development of liver cancer(Zhou et al. 2021). Many studies consider NOD2 as a potential risk factor that promotes the occurrence of cancer. However, autophagy is a cellular degradation process that involves the recycling of lysosome-mediated organelles and protein aggregates, as well as the destruction of intracellular pathogens. Autophagy and its regulatory mechanisms are involved in the homeostasis and repair of the intestine, supporting intestinal barrier function by regulating tight junctions and preventing cell death in response to cellular stress (Mizushima 2018). Research indicates that various natural products and their extracts can enhance autophagy, thus ameliorating the onset of IBD and CRC. Therefore, regulating the expression of NOD2 to suppress inflammatory responses and promote autophagy may represent an effective therapeutic approach for inhibiting IBD and combating CRC (Guo et al. 2014, 2016; Lee et al. 2011; Li et al. 2017; Yanai et al. 2016; Zhao et al. 2015).

Natural products constitute a vast treasure trove, with many components exhibiting varied effects at different concentrations. *Myristica fragrans Houtt*, commonly known as nutmeg, is a seed from an evergreen tree species, characterized as a natural plant with homologous medicinal and culinary properties ( http://www.worldfloraonline.org/tpl/kew-103019). *Nutmeg* has been widely used in traditional Chinese medicine, with its pharmacological effects aimed at treating gastrointestinal discomfort, abdominal bloating and pain, and persistent diarrhea(Schepetkin et al. 2016; Sepulveda et al. 2017). Dehydrodiisoeugenol (DEH) is a representative and major benzofuran-type neolignan found in *Myristica fragrans Houtt*, which has been demonstrated to possess antibacterial, anti-inflammatory, anticancer, and antioxidant effects (El-Alfy et al. 2009; Fujisawa et al. 2007; León-Díaz et al. 2013; Li et al. 2021). Previous studies have preliminarily found that DEH can inhibit the expression of cyclooxygenase-2 (COX-2) in macrophages stimulated by lipopolysaccharide (LPS), demonstrating certain anti-inflammatory activity(Murakami et al. 2005). However, the exact effects and molecular mechanisms of DEH in anti-IBD and anti-CRC are still unclear.

In this study, the main focus was to explore the dual therapeutic effects of DEH in anti-inflammation and anti-cancer using cellular and animal models, and elucidate its underlying mechanisms of action. LPS/IFNγ-stimulated RAW264.7 macrophages were used to establish an inflammation cell model to elucidate the anti-inflammatory effects of DEH. HCT116 cells were employed to elucidate the anti-cancer activity of DEH. Additionally, we further explored the potential mechanisms underlying the anti-inflammatory and anti-cancer activities mediated by DEH. Based on increasing research demonstrating the therapeutic effects of targeting NOD2 and autophagy in inflammation and CRC, this study for the first time investigated the efficacy of low concentrations of DEH in inhibiting IBD inflammation by suppressing NOD2, and high concentrations of DEH were found to promote NOD2-induced cell autophagy, exhibiting significant anti-CRC effects. This may provide scientific basis for natural product-based therapies against IBD and CRC. The research results indicate that DEH may be a promising therapeutic application for treating IBD and CRC.

## Materials and methods

### Chemical and regents

Dehydrodiisoeugenol (CAS:2680-81-1, purity ≥ 99.98%) was purchased by MedChemExpress (New Jersey, USA); Salicylazosulfapyridine (SASP, Cat#MB5634) was purchased by Meilun Biotechnology (Dalian, China). LPS extracted from E. coli 055:B5 (Cat# L6529) was obtained from Sigma Aldrich (Shanghai, China). Recombinant murine IFNγ (Cat# 50709-MNAH) was provided by MedChemExpress (New Jersey, USA). Curcumin (Cat# SC0299) was purchased from MedChemExpress (New Jersey, USA). Primary antibodies include against COX-2 (Cat# 12375-1-AP), iNOS(Cat# 18985-1-AP), α-Tubulin (Cat# 11224-1-AP), GAPDH (Cat# 10494-1-AP) and HRP-conjugated affinipure goat antibody (Cat# SA00001-2) were purchased from Proteintech (Wuhan, China). Primary antibodies against IKKα/β (Cat# AF2221), phospho-IKKα/β (p-IKKα/β) (Cat# AF5839), IκBα (Cat# AF1282), phospho-IκBα (p-IκBα) (Cat# AF 1870), ERK (Cat# AF1051), phospho-ERK (Cat# AF5818), JNK (Cat# AF1048), phospho-JNK (Cat# AF1762), p38 (Cat# AF7668), phospho-p38 (Cat# AF5887) were purchased from Beyotime (Shanghai, China). HCT116, HT29 and RAW264.7 cells were provided by Fuheng (Shanghai, China).

### Cell culture

RAW264.7, HCT116 and HT29 cells were cultured in high-glucose DMEM (Gibco, USA) supplemented with 10% fetal bovine serum (Gibco, USA) and 1% penicillin/streptomycin (Gibco, USA) under conditions of 37 °C and 5% CO2. Bone marrow derived macrophages (BMDMs) were extracted from C57BL/6 mice using a sterile syringe, BMDMs cultured in DMEM with 10% FBS and 1% Penicillin-Streptomycin, as well as supplemented with 50 ng/mL M-CSF.

### Cell viability assay

RAW264.7 cells (1.5 × 10^4^ cells/100 µl), HCT116 cells (1.0 × 10^4^ cells/100 µl) and HT29 cells (1.0 × 10^4^ cells/100 µl) were seeded in a 96-well plate. After being pretreated with different concentrations of DEH (0, 0.5, 1, 2, 4, 8, 10, 20 µM) for 2 h, RAW264.7 cells were stimulated with LPS (0.5 µg/ml) and IFNγ (10 ng/ml) for 24 h. HCT116 and HT29 cells were treated with different concentrations of DEH (0, 1, 2, 4, 8, 10, 20, 40, 80, 100 µM) for 24 h. Each well was supplemented with 100 µL of DMEM medium containing 10% CCK-8 reagent (Beyotime, Shanghai, China). The plate was then incubated at 37 °C in the dark for 1 h. The absorbance values at 450 nm for each well were measured using a microplate reader (FlexStation 3; Molecular Devices) to calculate cell viability.

### Measurement of nitric oxide (NO) production

RAW 264.7 cells (1.5 × 10^4^ cells/100µl) or BMDMs were seeded overnight in a 96-well plate, and treated with the positive control drug (curcumin, 5 µM) and DEH (2.5, 5, 10 µM), following stimulated by LPS (0.5 µg/ml) and recombinant IFNγ (10 ng/ml) for 24 h. Nitric oxide (NO) released into the cell supernatant was measured using Griess reagent (Beyotime, Shanghai, China) according to the manufacturer’s instructions, followed by immediate absorbance measurement at 540 nm.

### Measurement of prostaglandin E_2_ (PGE_2_) levels

RAW264.7 cells (1.0 × 10^6^ cells/ml) were seeded overnight in a 24-well plate, after being treated with the positive control drug (curcumin, 5 µM) and DEH (2.5, 5, 10 µM), following stimulated with LPS (0.5 µg/ml) and IFNγ (10 ng/ml) for 24 h. Enzyme-linked immunosorbent assay (ELISA) kits (R&D Systems, USA) were used to measure the concentration of PGE_2_ in the cell culture supernatant according to the manufacturer’s instructions.

### Western blotting assay

Added 1% protease inhibitor (1:100, Cat# K1007; ApexBio, Houston, USA) to RIPA buffer for total protein extraction from tissues and cells. Used the BCA protein assay kit (Cat# P0012S; Beyotime, Shanghai, China) to measure protein concentration. According to Bayorh et al. (Bayorh et al. 2006), nuclear and cytoplasmic proteins were extracted and separated using the nuclear and cytoplasmic protein extraction kit (Beyotime, Shanghai, China). The total protein amount used was 20 µg, separated by 10% sodium dodecyl sulfate-polyacrylamide gel electrophoresis (SDS-PAGE), and transferred onto polyvinylidene fluoride (PVDF, Thermo Fisher Scientific, America) membranes. Then blocked with 5% non-fat milk in Tris-buffered saline with 0.1% Tween-20 (TBS-T) for 1 h, incubated with primary antibody (diluted 1:1000) overnight at 4 °C. Washed the membrane three times with TBS-T, then incubated with secondary antibody (diluted 1:5000) at room temperature for 2 h. After being washed three times with TBS-T, the protein signal was observed using electrochemiluminescence (ECL) reagent (Cat# 4AW011, 4 A Biotech Company, Beijing, China), and quantitated using ImageJ software.

### Colony formation assay

HCT116 cells (1 × 10^3^ cells/1 ml) were seeded in a 6-well cell culture plate per well and culture for 10 days. Then fix the cells with 4% paraformaldehyde (PFA) for 15 min, stain with 0.5% crystal violet solution for 30 min, and wash three times with PBS. Count the colonies of cells when the plate is completely dry.

### Flow cytometry for cell cycle and apoptosis

Using the Cell Cycle Analysis Kit and Annexin V-FITC/PI Apoptosis Detection Kit (Beyotime, Shanghai, China) to detect cell cycle and apoptosis of HCT116 cells. HCT116 cells were seeded at 1 × 10^^6^ cells per well in a 6-well plate. After allowing cells to adhere, they were treated with different concentrations of DEH (0, 20, 40 µM) for 24 h. Collected cells and washed with pre-chilled PBS, then proceed with the experiment according to the instructions of Cell Cycle Analysis Kit and Annexin V-FITC/PI Apoptosis kit. Flow cytometer (CytoFLEX, Beckman) was used to analyze the distribution of HCT116 cells in G0/G1, S, or G2/M phases, as well as the occurrence of cell apoptosis. The data was analyzed using FlowJo version 10.8.1.

### Collection of human tissues

A total of 9 samples were collected (3 colon cancer tissues from patients who underwent operative treatment, 3 IBD tissues and 3 normal colon mucous membrane tissues from mucosal biopsy specimens) at the Affiliated Hospital of North Sichuan Medical College, between 2023.1 and 2023.12 from patients with no preoperative radiation or chemotherapy or drug treatment. This study was approved by the Ethics Committee of the Affiliated Hospital of North Sichuan Medical College (approval no. 2022ER439-1), and informed consent was obtained from all participants.

### Animals

SPF-grade male BALB/c mice, aged 6–8 weeks and weighing 20–22 g, were purchased from Chengdu Yaokang Biotechnology Co., Ltd. (Jiangsu, China). The mice were housed in plastic cages filled with aspen wood shavings, maintained on a 12-hour light/dark cycle at 25 ± 1 °C, and provided with standard food and water ad libitum. The mice were adaptively fed for 7 days. All experiments were approved by the relevant ethics regulations of Affiliated Hospital of North Sichuan Medical College (approval no. 2022ER439-1), in accordance with the Guidelines for the Care and Use of Laboratory Animals in Biomedical Research.

### IBD model establishment and group intervention

Randomly divide mice into 6 groups: Control group, DSS group, SASP group, DEH-low-dose (DEH-L) group, DEH-middle-dose (DEH-M) group, DEH-high-dose (DEH-H) group (5 mice/group). The DSS group, SASP group, DEH-L group, DEH-M group, and DEH-H group were administered 3.5% DSS dissolved in double-distilled water via gavage from day 2 to day 8. The SASP group (50 mg/kg/day), DEH-L group (5 mg/kg/day), DEH-M group (10 mg/kg/day), and DEH-H group (20 mg/kg/day) were administered via gavage from day 1 to day 8, once daily. The normal group and DSS group mice were administered an equivalent solvent (0.5% carboxymethyl cellulose sodium). Body weights were recorded from day 2 to day 8.

### Xenograft tumor growth experiments

In brief, BALB/c nude mice were subcutaneously injected with HCT116 cells (2 × 10^^6^ cells) into their bodies. About one week later, the successfully transplanted mice were randomly divided into the control group and the DEH-40 mg/kg group. Then, the control group received intraperitoneal injection of 100µL PBS (containing 0.1% DMSO), while the mice in the DEH group were orally gavaged with DEH at 40 mg/kg once daily for 20 consecutive days. Tumor sizes and body weights were measured and recorded every 5 days. The mice were euthanized, and tumors were excised, photographed, and weighed. Tumor volume was calculated using the formula: Volume = 0.5 × (length × width^^2^). Additionally, some tumor tissues were fixed in 4% paraformaldehyde for further experiments.

### Histopathological examination

Two hours after the last dose, the mice were euthanized at the site of cervical dislocation. Subsequently, colon tissues were isolated to measure their lengths. The colon tissues were then fixed overnight in 4% paraformaldehyde, followed by embedding in paraffin. After hematoxylin and eosin (HE) staining, pathological examination of the specimens was conducted under a microscope.

### Enzyme-linked immunosorbent assay

Took colonic tissue, weighed same amount of sample and cuted them into small pieces, then homogenized the samples into 10% tissue homogenate using pre-chilled PBS at 4 °C, centrifuged at 13,000 rpm for 10 min at low temperature, then collected the supernatant, the concentration of MPO in the sample tissue was determined using the MPO assay kit (Nanjing Jiancheng Bioengineering Institute, Nanjing, China) according to the instructions. The levels of TNF-α, IL-6, and IL-1β in the serum were detected by ELISA kit (Valukine, R&D, Minneapolis, United States) according to the manual instuction.

### Immunohistochemistry

The tissues were fixed in 4% paraformaldehyde at 4 °C for 24 h. After washing with PBS, the samples were dehydrated, embedded in paraffin, and then sectioned. The paraffin sections were rehydrated and deparaffinized, followed by a 5-minute incubation in 3% hydrogen peroxide. After washing three times with distilled water, the sections were then subjected to antigen retrieval in at room temperature, the sections were blocked with 5% goat serum for 1 h, followed by overnight incubation at 4 °C with the one-step tunel staining reagent.er (pH 6.0). At room temperature, the sections were blocked with 5% goat serum for 1 h, followed by overnight incubation at 4 °C with the one-step tunel staining reagent, ki67 Mouse mAb (1:200) or LC3B Rabbit mAb (1:100). After washing with PBS, the sections were incubated with biotinylated goat anti-mouse IgG at 37 °C for 2 h. Following staining with DAB reagent, amplified positive signals were observed under a microscope.

### Periodic Acid-Schiff (PAS) staining

PAS staining was performed using the Schiff’s reagent kit with periodic acid according to the manufacturer’s instructions. After dewaxing the sections, incubation with periodic acid was carried out at room temperature for 10 min, followed by rinsing with distilled water. Then, the sections were stained with Schiff’s reagent at 37 °C for 30 min and rinsed with distilled water. Subsequently, staining was performed with Hematoxylin S for 30 s at 37 °C, followed by two rinses with distilled water. After dehydration, the sections were sealed with neutral balsam on slides. All images were captured using an X71 (U-RFL-T) microscope at 400x magnification. The mean of the integrated optical density was analyzed using ImageJ software (NIH, United States).

### Statistical analysis

All experiments were carried out at least three times independently. Data were presented as the mean ± SEM Statistical significance (^ns^*P* ≥ 0.05, ^*^P < 0.05, ^**^P < 0.01, and ^***^P < 0.001) was analyzed by GraphPad Prism 9.0 using one-way ANOVA or Student’s t-test. Each experiment was independently repeated 3 times at least.

## Results

### Dehydrodiisoeugenol inhibited the inflammatory response of macrophages stimulated by LPS/IFNγ

**Fig. 1:**
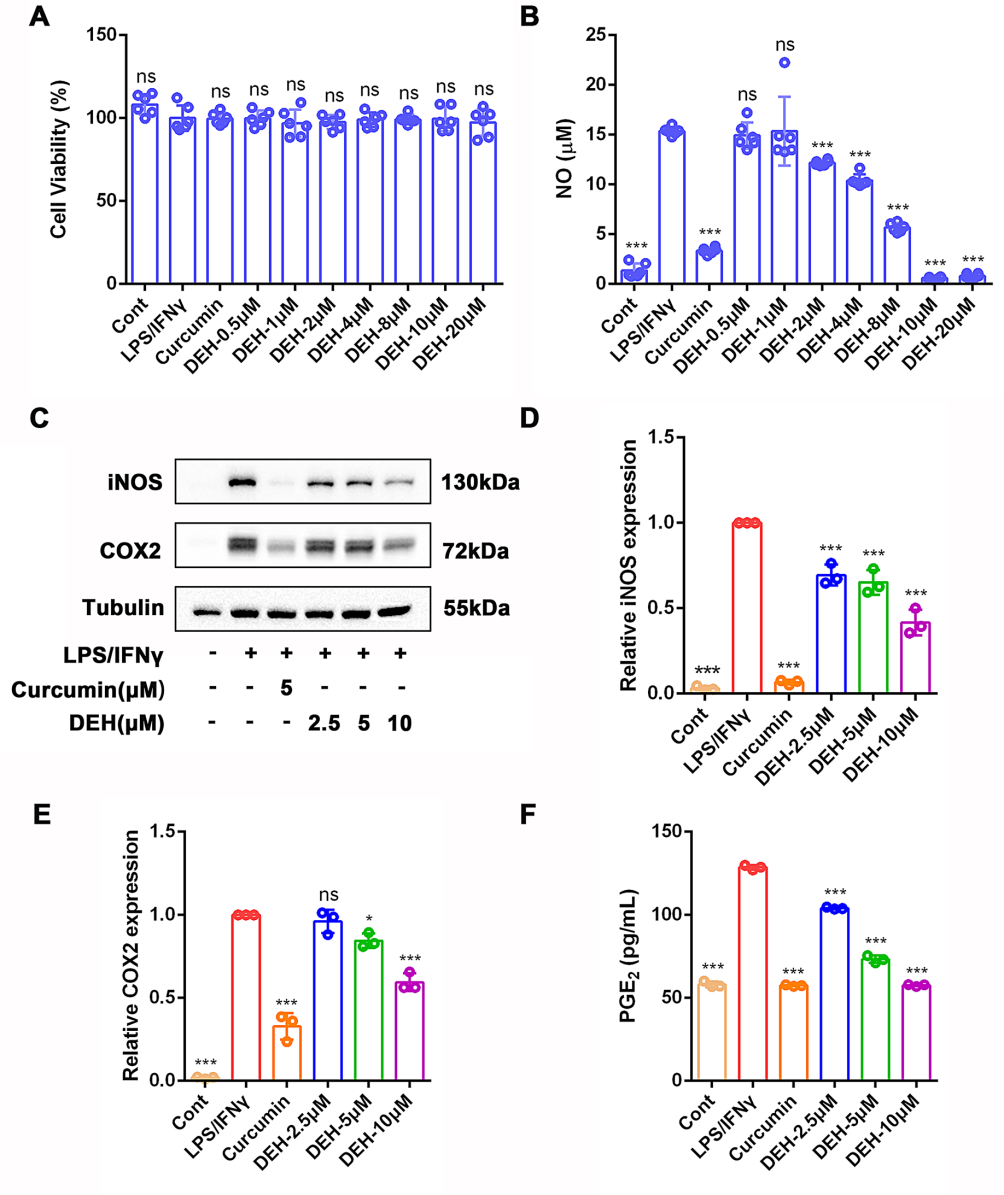
DEH suppresses inflammation in LPS/IFNγ-stimulated RAW264.7 cells in vitro. (**A**)The CCK-8 assay to determine the cell viability of LPS (0.5 µg/ml)/IFNγ (10ng/ml) stimulated RAW264.7 cells treated with different concentration of DEH or Curcumin (5µM). (**B**)The effect of DEH on NO production in LPS (0.5 µg/ml)/IFNγ (10ng/ml) stimulated mouse RAW264.7 macrophage cell line. (**C**) Western blotting analysis of iNOS and COX2 protein expression in RAW264.7 cells treated with different concentrations of DEH or curcumin (5µM) following LPS/IFNγ stimulation. (**D**-**E**) Density analysis of iNOS (**D**) and COX-2 (**E**), normalized to β-actin. (**F**) The effect of DEH on PGE_2_ release in LPS/IFNγ-induced RAW264.7 cells. The data are presented as mean ± SEM, *n* = 3. * *p*<0.05, ** *p*<0.01, **** *p*<0.001. Compared to LPS/IFNγ

Due to the critical role of macrophages in the inflammatory process, this study utilized LPS/IFNγ-stimulated RAW264.7 macrophages to establish an in vitro model of ulcerative colitis (UC) and evaluated the anti-inflammatory effects of DEH in vitro. The research results indicated that different concentrations of DEH showed no significant cytotoxicity against LPS/IFNγ-induced RAW264.7 cells (Fig. 1A). In addition, the production of NO after being stimulated by LPS/IFNγ is an important indicator of the inflammatory process. As expected, DEH can inhibit the production of NO in LPS/IFNγ-stimulated RAW264.7 cells in a dose-dependent manner, with more significant effects observed at 10 µM and 20 µM (Fig. 1B), this result was also validated in BMDM cells (Supplementary Fig. 1A). Furthermore, the results indicated that treatment with DEH (2.5 µM, 5 µM, 10 µM) or curcumin (5 µM) significantly inhibits the expression of iNOS and COX2. The inhibitory effect of DEH at 10 µM is most pronounced (Fig. 1C-E). PGE_2_ is another important inflammatory mediator. We further examined the expression of PGE_2_ in LPS/IFNγ-treated RAW264.7 cells. As shown in Fig. 1F, treatment with DEH (2.5µM, 5µM, 10µM) or curcumin (5µM) significantly reduced the release of PGE_2_. In addition, DEH can inhibit the production of inflammatory factors, such as IL-1β, IL-6, and TNFα in RAW264.7 cells stimulated by LPS/IFNγ (Supplementary Fig. 1B-1D). All the results showed in above suggests that DEH exerts a pronounced anti-inflammatory effect on LPS/IFNγ-stimulated RAW264.7 macrophages.

### Dehydrodiisoeugenol suppressed the activation of NF-κB and MAPK signaling pathways in LPS/IFNγ-stimulated RAW264.7 cells

**Fig. 2:**
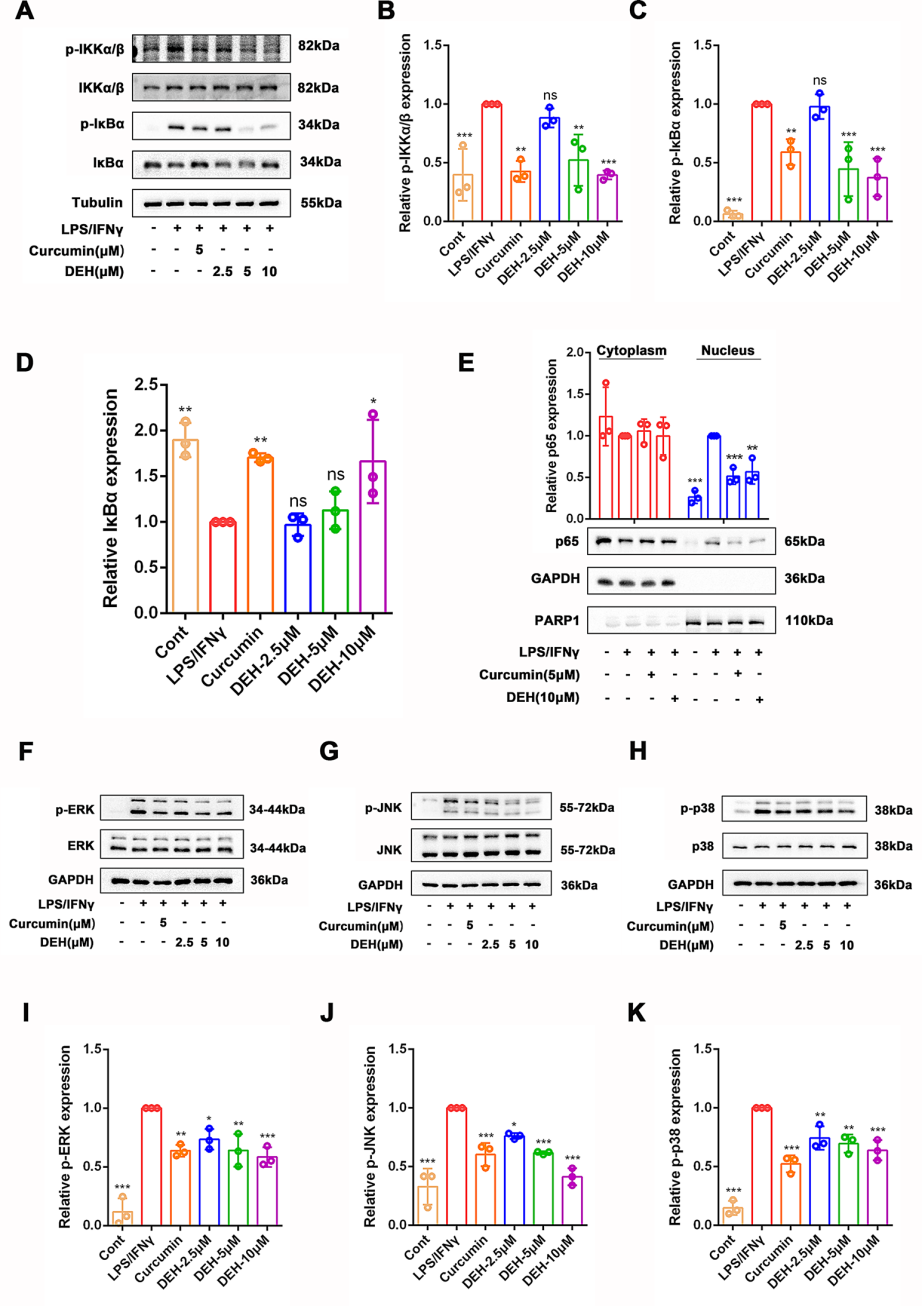
DEH prevents RAW264.7 cells from inflammation stimulated by LPS/IFNγ through inhibiting NF-κB and MAPKs signaling pathway. (**A**) LPS/IFNγ-induced RAW264.7 cells were treated with DEH (2.5µM, 5µM, 10µM) or curcumin (5µM) for 24 h, and the expression levels of p-Ikkα/β, Ikkα/β, p-IκBα, and IκBα were detected using western blotting. Tubulin was used as a reference. (**B**-**D**) The semi-quantitative levels of p-Ikkα/β, Ikkα/β, p-IκBα, and IκBα following DEH treatment compared to LPS/IFNγ stimulation. (**E**) Western blotting was used to detect the expression levels of p65 and PARP1 in the cell nucleus and cytoplasm. Using GAPDH as a reference. (**F**-**H**) Western blotting to detect the phosphorylation levels of ERK, JNK, and p38. (**I**-**K**) Density measurements were analyzed for p-ERK (**I**), p-JNK (**J**), and p-p38 (**K**), normalized against total ERK, JNK, and p38 proteins. The data are presented as mean ± SEM, *n* = 3. * *p*<0.05, ** *p*<0.01, **** *p*<0.001. Compared to LPS/IFNγ

NF-κB is a key transcription factor involved in immune and inflammatory processes, and its overactivation plays a crucial role in the onset and progression of ulcerative colitis (UC)(Tambuwala 2016). Therefore, we investigated whether the anti-inflammatory activity of DEH depends on its regulation of the NF-κB signaling pathway. Under stimulation with LPS/IFNγ, phospho-IKKα/β, IκBα, and phospho-IκBα levels were increased in RAW264.7 cells (Fig. 5A-C), promoting degradation of IκBα (Fig. 5D). Different concentrations of DEH or curcumin (5µM) were able to inhibit the phosphorylation of Ikk and IκBα induced by LPS/IFNγ. However, DEH did not show a significantly anti-inflammatory effect at the concentration of 2.5 µM. It is noteworthy that DEH at 10µM or curcumin at 5µM can inhibit the protein degradation of IκBα induced by LPS/IFNγ (Fig. 2A-D). In addition, treatment with LPS/IFNγ also increased the level of NF-κB p65 subunit in the nucleus, promoting nuclear translocation of p65 (Fig. 5E), indicated the activation of the NF-κB signaling pathway. Treatment with DEH or curcumin (5µM) effectively inhibits the nuclear translocation of NF-κB p65 in LPS/IFNγ-induced RAW264.7 cells (Fig. 5E). The above results indicated that DEH is an effective NF-κB signaling inhibitor in LPS/IFNγ-stimulated RAW264.7 macrophages.

MAPKs, as crucial extracellular signal transduction pathways, is another key signaling pathway regulating inflammatory responses and is involved in the pathogenesis of UC(Ma et al. 2021). ERK regulates cell proliferation and differentiation, JNK is involved in cellular responses to radiation, osmotic stress, and temperature changes, and p38 plays a role in inflammation and cell apoptosis, and is targeted for the development of anti-inflammatory drugs(Yan et al. 2022). Therefore, we investigated whether DEH affects the activation of the MAPK signaling pathway. The phosphorylated levels of ERK, JNK, and p38 were significantly increased in LPS/IFNγ-stimulated RAW264.7 cells. Different concentrations of DEH (2.5µM, 5µM, 10µM) or curcumin (5µM) significantly inhibited LPS/IFNγ-induced MAPK signaling activation (Fig. 2F-K). In summary, DEH exhibits a potently inhibitory effect on the activation of MAPKs signaling pathway in LPS/IFNγ-stimulated RAW264.7 macrophages.

### Oral administration of DEH conferred protective effects against DSS-induced colitis in mice

**Fig. 3:**
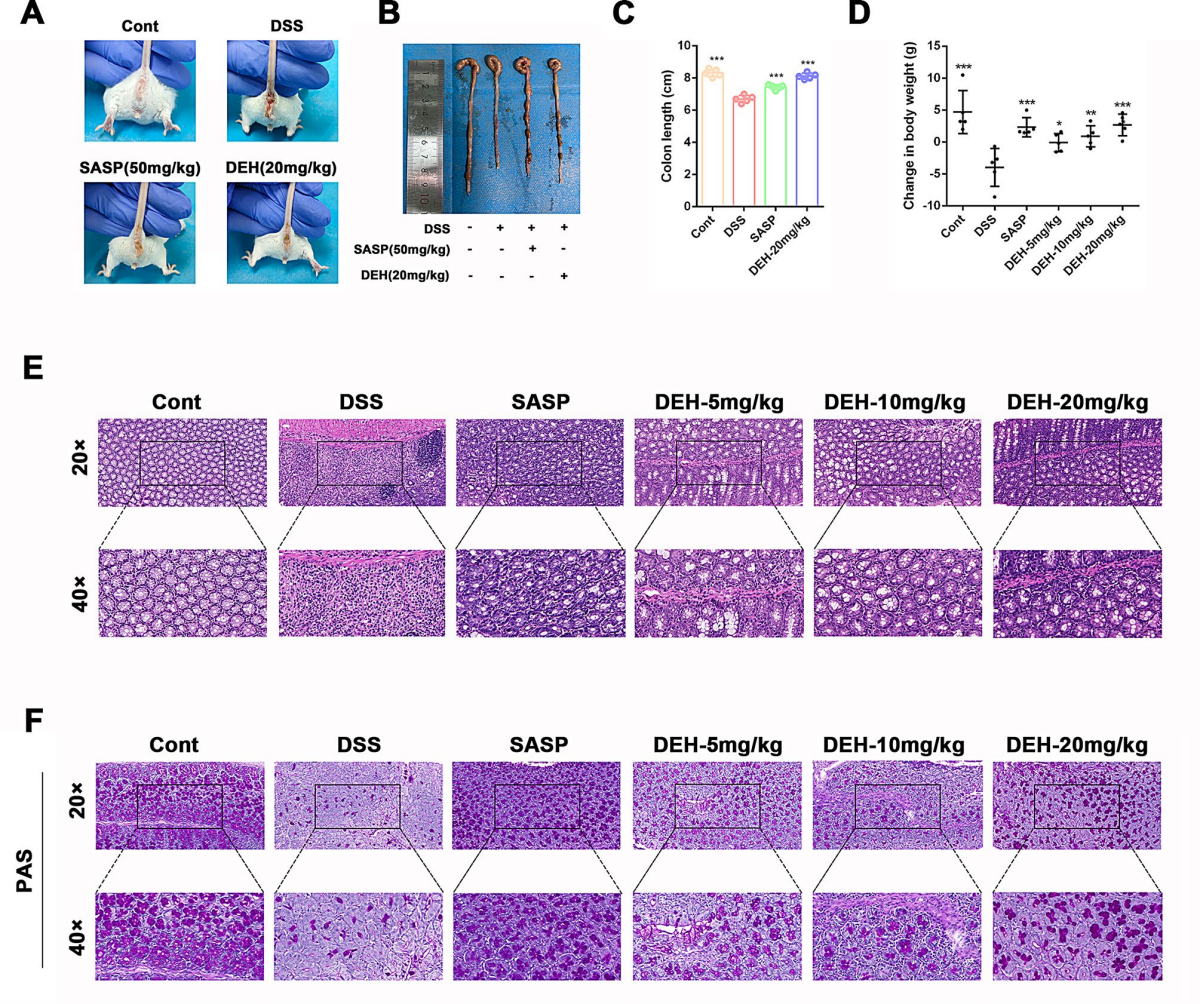
DEH alleviates colitis subjected to DSS in vivo. Oral administration of DEH inhibits inflammation in DSS-induced colitis in mice. (**A**) Representative pictures of the colons surrounding control, DSS, SASP, and DEH groups of mice. Representative pictures of colons from mice exposed to DSS (**B**) and statistical data on colon length (**C**), as well as the effect of DEH on body weight changed in mice (**D**). Representative images of colon tissue stained with H&E (**E**) and PAS (**F**) for each group, 20× or 40×. Data are represented as mean ± SEM, *n* = 5. *p < 0.05, **p < 0.01, ***p < 0.001, ****p < 0.0001. Compared to DSS group

To assess the therapeutic effect of DEH on UC, we established colitis mice induced by DSS in vivo. Perianal observations indicated that treatment with DEH (20 mg/kg) or SASP (50 mg/kg) significantly improved rectal bleeding induced by DSS compared to the model group (Fig. 3A). The colon length of mice in the normal group was 9.14 ± 0.52 cm, whereas in the model group it was 7.68 ± 0.52 cm, indicating that DSS treatment could lead to colon shortening. However, after DEH intervention, the colon length of mice showed a significant increase compared to the model group (Fig. 3B). As shown in Fig. 3C, mice in the control group exhibited stable body weight. In contrast, mice in the other groups that consumed 3.5% DSS displayed weight loss. In addition, compared to the model group, mice in the DEH (5, 10, 20 mg/kg) or SASP (50 mg/kg) groups also showed a significant increase in body weight (Fig. 3C). Subsequently, we performed HE staining (Fig. 3D) and PAS staining (Fig. 3E) on colon tissues. The model group exhibited severe mucosal damage and edema in the colon, with loss of goblet cells and crypts, and significant infiltration of inflammatory cells. Administration of DEH (5, 10, 20 mg/kg) or SASP (50 mg/kg) resulted in varying degrees of alleviation of colonic tissue symptoms. There was recovery of mucosal epithelial damage, relatively intact colonic tissue, and reduced infiltration of inflammatory cells. In summary, DEH significantly improves colonic inflammation symptoms in DSS-induced mice colitis and exhibits a notable inhibitory effect on the development of UC.

### DEH improved intestinal epithelial barrier damage and intestinal inflammation response induced by DSS in mice

**Fig. 4:**
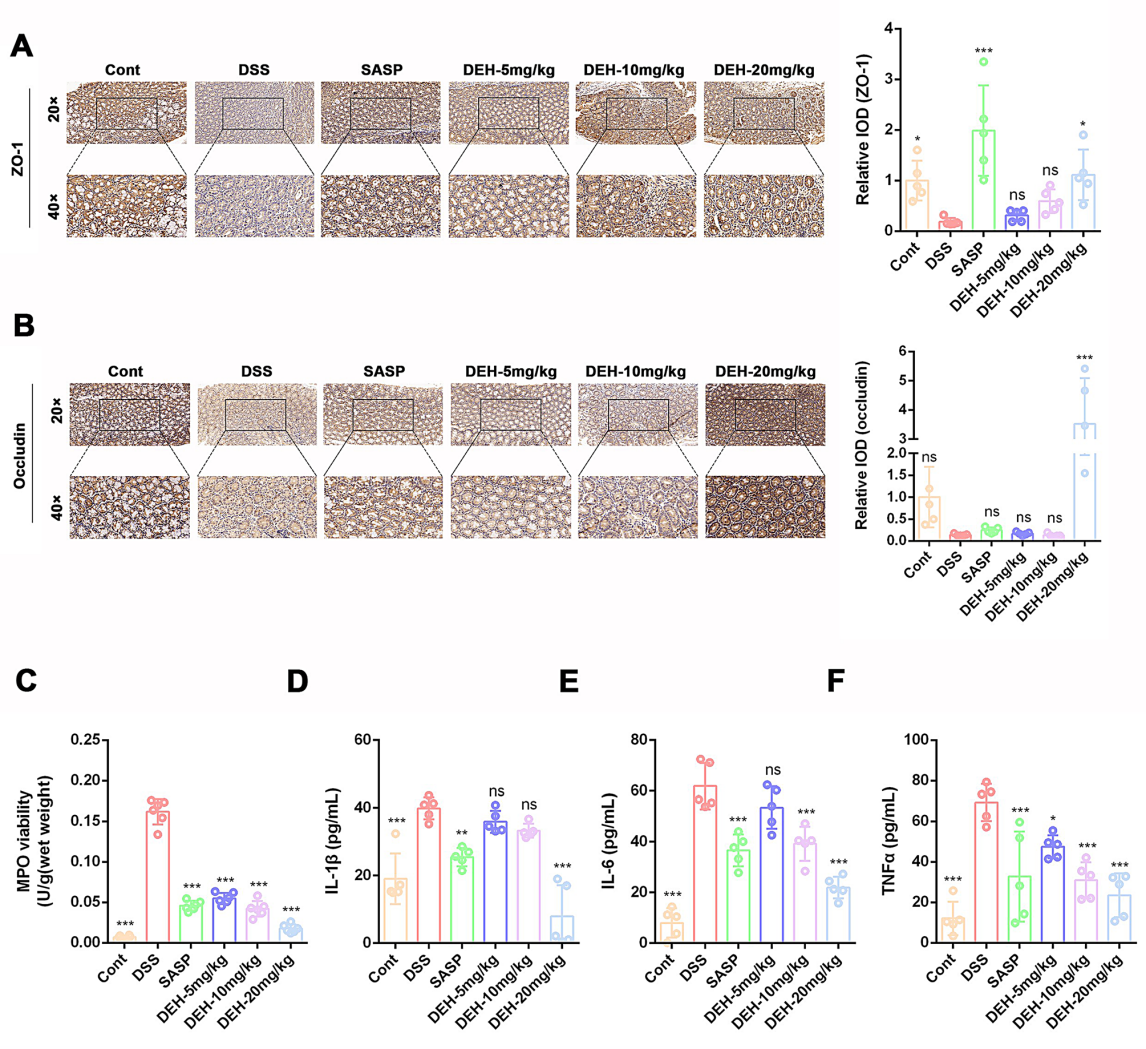
DEH attenuates inflammatory response involved in DSS-induced colitis. Representative pictures (**A**) and statistical data (**B**) showing the expression of ZO-1 and Occludin in colonic tissues of mice from the control group, DSS group, SASP group, and DEH group. (**C**-**F**) ELISA was used to measure the expression levels of MPO, IL-1β, TNF-α, and IL-6 In each group. Data are represented as mean ± SEM, *n* = 5. *p < 0.05, **p < 0.01, ***p < 0.001, ****p < 0.0001. Compared to DSS group

The impairment of intestinal mucosal mechanical barrier function leading to increased intestinal permeability is an important pathogenic mechanism in UC. Promoting mucosal healing and repairing the damaged intestinal mucosal barrier are also key aspects of UC treatment(Roberts-Thomson et al. 2019). Therefore, we assessed the effects of DEH on the intestinal mucosal barrier in DSS-induced colitis. We conducted immunohistochemical staining for ZO-1 and Occludin in colonic tissues. The results showed a significant decrease in ZO-1 and Occludin protein expression in the colonic tissues of DSS-treated mice, indicating compromised barrier function and increased intestinal mucosal permeability during the UC pathological process. Treatment with DEH (20 mg/kg) or SASP (50 mg/kg) significantly restored the loss of ZO-1 protein (Fig. 4A), whereas only DEH (20 mg/kg) intervention increased Occludin expression (Fig. 4B). This helps to reduce intestinal mucosal permeability and protect the mechanical barrier of the intestinal mucosa.

Several types of immune cells, including macrophages and neutrophils, are activated during the active phase of UC and recruited into intestinal tissues. These activated immune cells produce excessive pro-inflammatory cytokines, contributing to inflammation and colonic damage(Geremia et al. 2014). Treatment with DSS significantly increased levels of specific markers for neutrophils such as MPO, as well as levels of TNF-α, IL-6, and IL-1β in the colon. Following treatment with DEH (5, 10, 20 mg/kg) or SASP (50 mg/kg), the levels of these factors and proteins mentioned above showed a significant decrease (Fig. 4C-F). The results indicate that DEH dose-dependently reduces levels of TNF-α, IL-6, IL-1β, and MPO in UC mice. These results suggest that DEH affected the expression of proteins related to intestinal mucosal barrier function, improves DSS-induced damage to the intestinal mucosal barrier in mice, and exhibits significant anti-inflammatory effects.

### DEH induced apoptosis in LPS/IFNγ-stimulated cells, inhibiting tumor cell invasion and migration

**Fig. 5:**
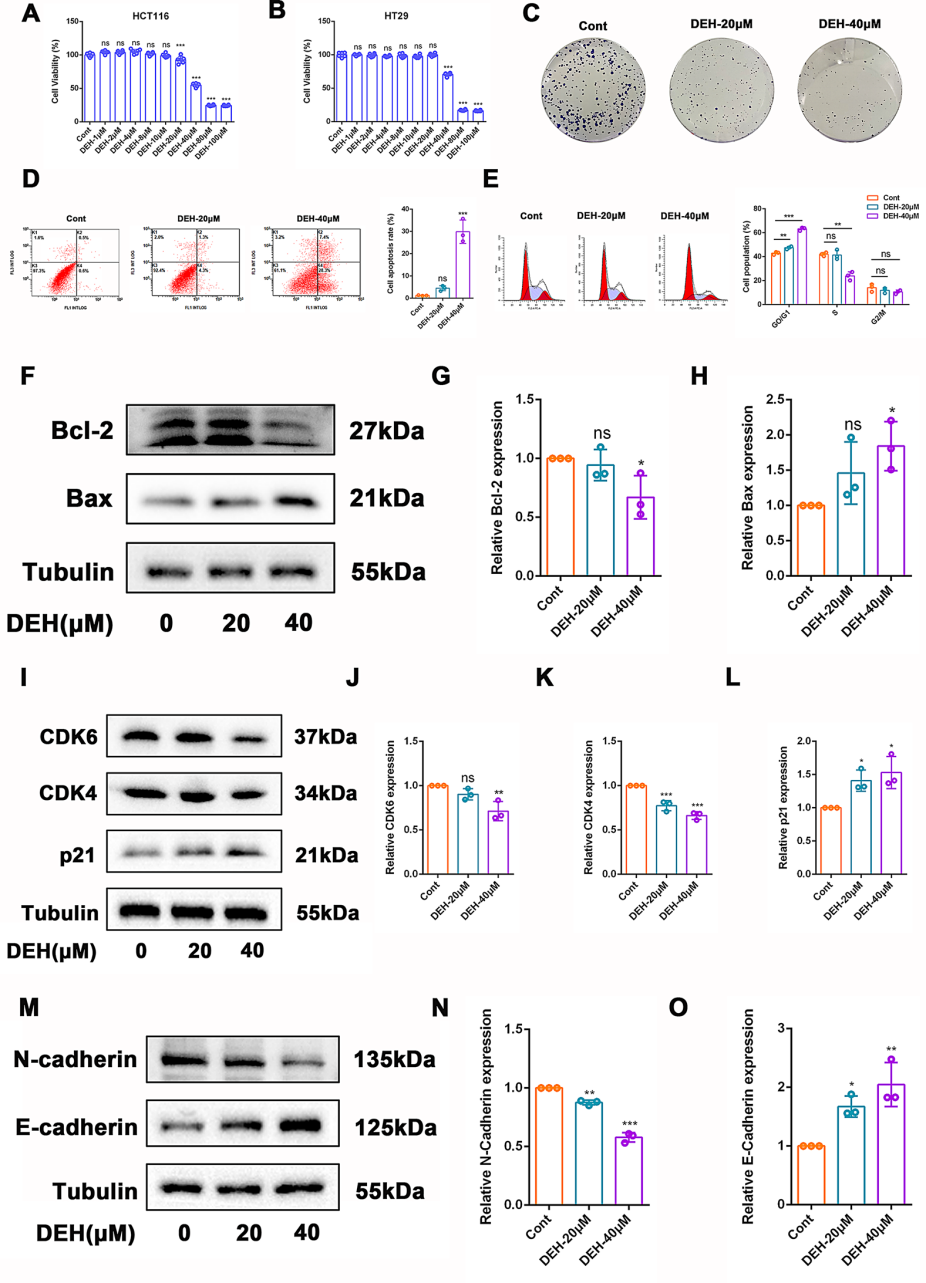
DEH inhibits colon cancer cells growth in vitro. (**A**-**B**) Using the CCK-8 assay to determine cell viability of HCT116 and HT29 cells treated with different concentration of DEH or Curcumin. (**C**) Here are representative images showing colony formation. HCT116 cells were incubated with DEH (20µM, 40µM) for 12 h, followed by a clonogenic assay. (**D**-**E**) Flow cytometry was used to detect apoptosis in the cells. HCT116 cells were treated with DEH (20µM, 40µM) for 24 h. (**F**) HCT116 cells were treated with DEH (20µM, 40µM) for 24 h. The expression levels of Bcl-2 and Bax were detected using western blotting. (**G**-**H**) The relative quantitative level of Bcl-2 and Bax. (**I**) HCT116 cells were treated with DEH (20µM, 40µM) for 24 h, and the expression levels of CDK6, CDK4, and p21 were detected using western blotting. (**J**-**L**) The relative quantitative level of CDK6, CDK4, and p21. (**M**) HCT116 cells were treated with DEH (20µM, 40µM) for 24 h, and the expression levels of N-cadherin and E-cadherin were detected using Western blotting. (**N**-**O**) The relative quantitative level of N-cadherin and E-cadherin. Tubulin was used as a reference. Data are represented as mean ± SEM, *n* = 3. *p < 0.05, **p < 0.01, ***p < 0.001, ****p < 0.0001. Compared to control group

We evaluated the anti-inflammatory effects of DEH using LPS/IFNγ-stimulated HCT116 and HT29 cells in vitro. Cell viability of the stimulated cells treated with different concentrations of DEH or curcumin was measured using the CCK-8 assay. The results showed that DEH (40, 80, 100 µM) significantly inhibited the viability of HCT116 and HT29 cells (Fig. 5A-B). Interestingly, DEH significantly inhibits the viability of HCT116 cells at 20 µM, while no significant inhibitory effect on HT29 cells at this concentration. This suggested that HCT116 cells are more sensitive to DEH compared to HT29 cells. Therefore, HCT116 cells were selected for further in vitro studies on the anti-colorectal cancer effects of DEH. As shown in Fig. 5C, compared to the control group, cells treated with DEH exhibit slower proliferation rates and form fewer cell colonies, indicating that DEH directly impacts the proliferation of HCT116 cells in vitro. Inducing apoptosis in cells is a common approach for treating tumors. Flow cytometry was used to detect apoptosis in colorectal cancer cells, and the results indicate a significant increase in apoptosis rates after treating HCT116 cells with DEH (20, 40 µM) for 24 h (Fig. 5D-E). The Bcl-2 family plays a crucial role in anti-apoptosis(Craig 1995). Interestingly, Western blot analysis indicated that treatment with DEH (40 µM) also suppresses the expression levels of Bcl-2 and upregulates the expression levels of Bax (Fig. 5F-H). Therefore, the Bcl-2 family is also one of the main reasons why DEH induces apoptosis in cells.

CDK4 and CDK6 play pivotal roles in the G1/S transition of the cell cycle. The primary mechanism of action of CDK4/6 inhibitors in anti-tumor activity is to induce cell cycle arrest. p21 acts as a regulator of cyclin-dependent kinases (CDKs) in the cell cycle by binding to and inhibiting cyclin-CDK1, CDK2, CDK4, and CDK6 complexes, thereby arresting the cell cycle progression in the G1 and S phases(Bertoli et al. 2013; Georgakilas et al. 2017). In this study, we found that treatment with 40µM DEH effectively reduced the protein levels of CDK6 and CDK4 (Fig. 5I-K). Furthermore, intervention with DEH at 20µM and 40µM increased the protein expression levels of p21(Fig. 5I and L), which plays a critical role in inhibiting cancer cell proliferation and invasion.

The metastasis of colorectal cancer cells is one of the main reasons for the high mortality rate of colorectal cancer, and epithelial-mesenchymal transition (EMT) is one of the primary mechanisms of tumor migration. As a promoter of tumor invasion, the loss of E-cadherin disrupts cell-cell adhesion(Nagy et al. 2022). In our experiment, we found that DEH (20, 40 µM) effectively promoted the expression of E-cadherin and decreased the expression of N-cadherin (Fig. 5M-O), thereby inhibiting tumor invasion and migration.

### DEH inhibited the growth of colon cancer

**Fig. 6:**
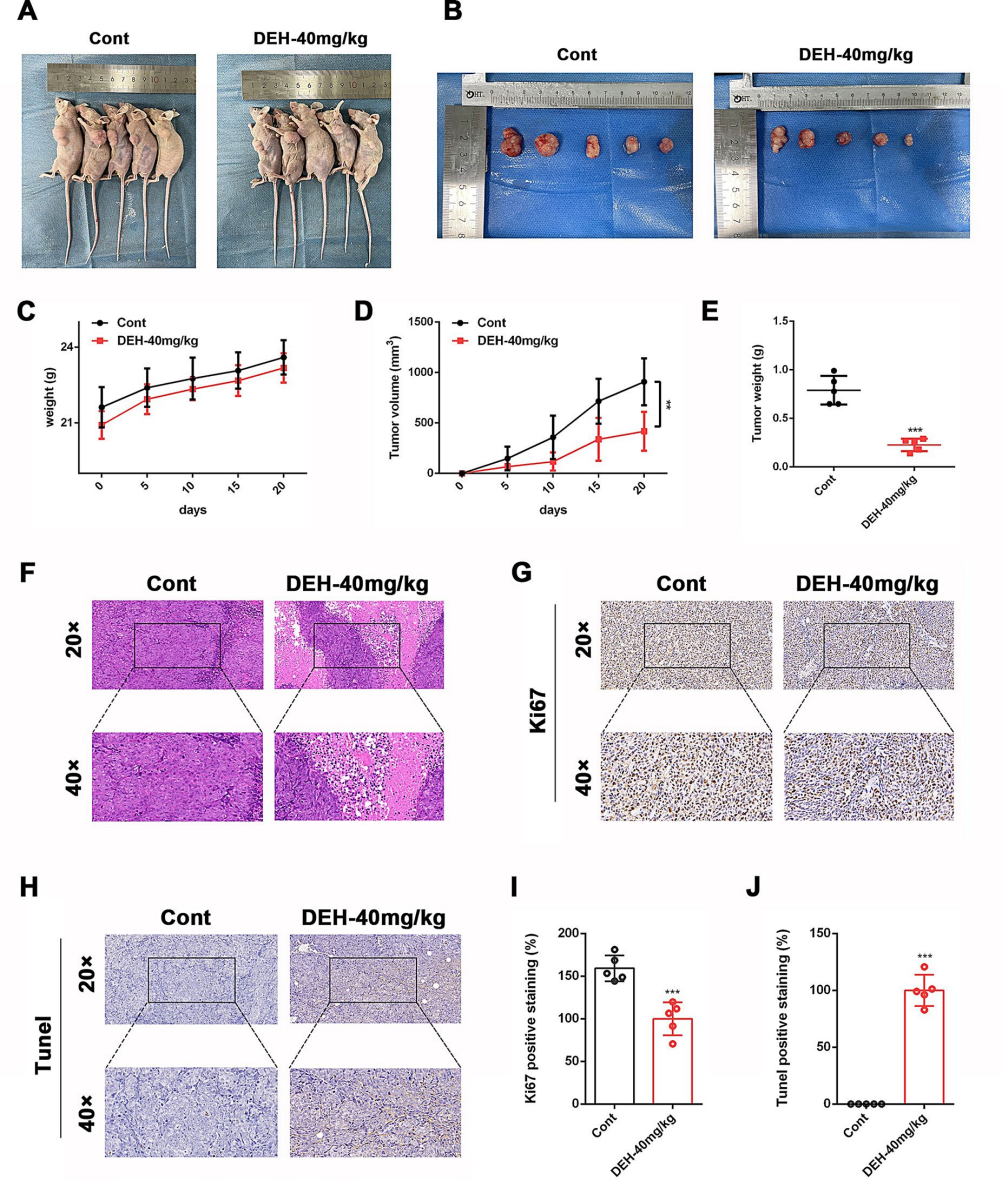
DEH inhibits xenografts growth in vivo. (**A**-**B**) Observing tumor growth in nude mice in the DEH (40 mg/kg) group compared to the control group. After successful tumor transplantation in nude mice, DEH (40 mg/kg) or an equivalent amount of solvent was administered orally for 20 days. Tumors were then collected for analysis. (**C**) Monitor the weight of nude mice every 5 days for a total duration of 20 days. (**D**-**E**) Measure the volume and weight of the transplanted tumor. The volume calculation formula is: Volume = 0.5 × (length × width²). (**F**) Apply the HE staining method to detect the effects of DEH (40 mg/kg) or an equivalent solvent gastric lavage on the nude mouse xenografts. Capture representative images at magnifications of ×20 and ×40. Perform Ki67 (**G**) and Tunel (**H**) staining on nude mouse xenografts treated with DEH (40 mg/kg) or an equivalent solvent gastric lavage. Capture representative images at magnifications of ×20 and ×40. In the tumor tissue, brown dots in the TUNEL staining represent apoptotic cells. (**I**-**J**) Quantitative data from Ki67 and TUNEL staining. Data are represented as mean ± SEM, *n* = 5. *p < 0.05, **p < 0.01, ***p < 0.001, ****p < 0.0001. Compared to control group

Through the above experiments, we have confirmed the significant anti-inflammatory and anti-tumor activities of DEH. Therefore, we further conducted subcutaneous transplantation of HCT116 cells into BALB/c nude mice. The treatment group received oral administration of DEH (40 mg/kg), while the control group received an equivalent amount of solvent injection simultaneously. After the experiment concluded, nude mice and tumor tissues were as shown in Fig. 6A and B. There was no significant difference in average weight between the two groups of mice (Fig. 6C). However, tumor weight and volume were significantly smaller in the DEH (40 mg/kg) treated group compared to the control group (Fig. 6D and E). Clearly, DEH can inhibit the growth of colon cancer in vivo. Subsequently, we performed HE staining on tumor tissues, Ki67 immunohistochemistry, and TUNEL staining. Consistent with in vitro experiments, treatment with DEH (40 mg/kg) promoted tumor necrosis (Fig. 6F). Ki67 is widely utilized as a proliferation marker in cancer histopathology, indicative of cells entering the later stages of the cell cycle, reaching its peak during mitosis(Sobecki et al. 2017). Therefore, this study distinguishes factors affecting tumor growth rate by assessing proliferation using Ki67. The expression levels of Ki67, an important marker of cell proliferation, were significantly lower than those in the control group (Fig. 6G and I). TUNEL analysis showed an increase in apoptotic cell numbers in the DEH (40 mg/kg) treatment group (Fig. 6H and J). Overall, DEH exhibits a significant inhibitory effect on colorectal cancer growth in vivo.

### DEH promoted autophagy in CRC cells

**Fig. 7:**
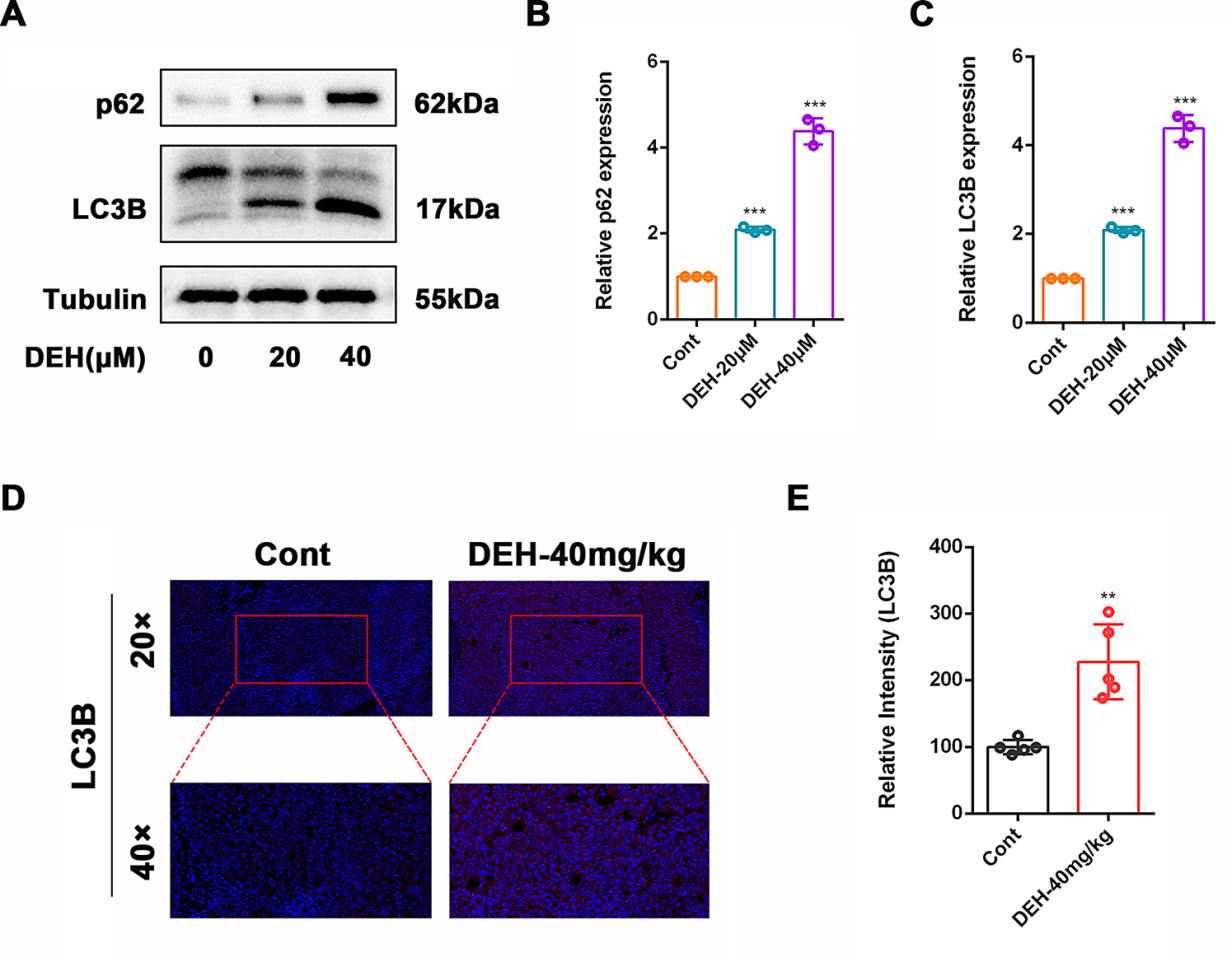
DEH promotes autophagy in xenografts. (**A**) Western blot was used to detect the expression of p62 and LC3B proteins following treatment with DEH (20µM, 40µM) for 24 h. Tubulin was used as a reference. (**B**-**C**) Semi-quantitative levels of p62 and LC3B. DEH treatment vs. control. (**D**) Typical fluorescence images of LC3B in tumor of xenografts are shown. After DEH (40 mg/kg) treatment, where blue fluorescence represents cell nuclei and red fluorescence represents LC3B. (**E**) The relatively quantitative fluorescence intensity of LC3B in tumor was analyzed using Image J. Data are represented as mean ± SEM, *n* = 5. *p < 0.05, **p < 0.01, ***p < 0.001, ****p < 0.0001. Compared to control group

Next, we aim to determine the anti-tumor response mediated by DEH through the autophagy pathway.LC3B and p62 are both markers of autophagy, with p62 protein being consumed as it directly participates in the autophagy process. In the same cell line, the higher the level of autophagy, the lower the levels of p62 detected within the cells. As an RNA-binding protein, LC3B can trigger rapid mRNA degradation during the autophagy process. The Western blotting result indicated that treatment with DEH (20 and 40 µM) for 24 h significantly increased the expression of p62 and LC3B (Fig. 7A-C). Immunofluorescence results also demonstrated a significant increase in LC3B expression in tumor tissues of mice treated with DEH (40 mg/kg). These findings suggest that autophagy signaling is involved in the anti-tumor effects of DEH.

### DEH targeted NOD2 to exert anti-inflammatory bowel disease (IBD) and anti-colorectal cancer (CRC) effects

NOD2 can influence cellular apoptosis in inflammatory responses through AMPK(Ma et al. 2020), NF-κB(Cui et al. 2021), and other pathways(Buteyn et al. 2020; Zhang et al. 2021). Considering that in vivo tumor experiments differ from the flat surfaces used in cell experiments, there may be differences in signal transduction processes. Through immunohistochemical analysis, we observed a significant increase in NOD2 expression in human UC tissues, while its expression was decreased in CRC tissues (Fig. 7A-B). In DSS-induced colitis mice, treatment with DEH (10, 20 mg/kg) or SASP (50 mg/kg) significantly reduced NOD2 protein levels (Fig. 7C-D).

**Fig. 8:**
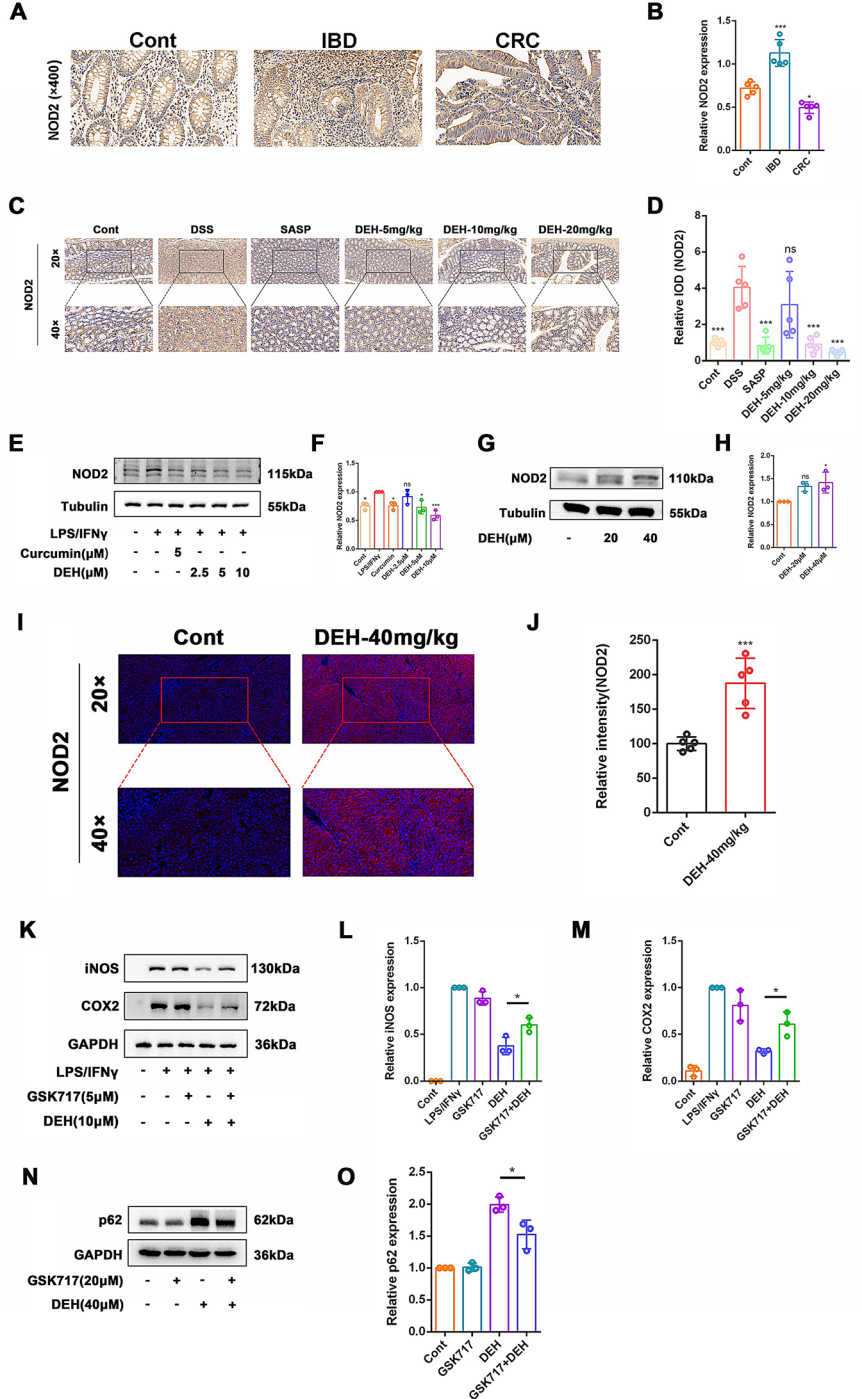
NOD2 plays a vital role in the anti-inflammatory response and anti-tumor growth of DEH. (**A**) Immunohistochemistry (IHC) method for detecting NOD2 expression in normal, IBD and CRC people. (**B**) Quantitative analysis of NOD2 expression levels. (**C**) IHC method was used to detect the protein expression of NOD2 in DSS-induced mice from different groups. Representative images were captured at magnifications of ×20 and ×40. (**D**) Quantitative analysis of the expression level of NOD2. DEH treatment vs. DSS. (**E**) Treatment with DEH (2.5µM, 5µM, 10µM) for 24 h, western blotting was used to detect the expression levels of NOD2. Tubulin was used as a reference. (**F**) The semi-quantitative levels of NOD2. DEH treatment vs. LPS/IFNγ. (**G**) Western blotting was used to detect the protein expression of NOD2. HCT116 cells were treated with DEH (20µM, 40µM) for 24 h, and the expression levels of NOD2 were assessed by western blotting. Tubulin was used as a reference. (**H**) Semi-quantitative levels of NOD2. DEH treatment vs. control. (**I**) Typical fluorescence images of NOD2 in xenografts tissue are shown. Blue fluorescence represents cell nuclei, and red fluorescence represents NOD2. (**J**)Analyzing tissue NOD2 using Image J. (**K**) RAW264.7 cells were treated with DEH (10µM) in the presence or absence of GSK717 (5µM) for 24 h, and the expression levels of iNOS and COX2 were detected using Western blotting. GAPDH was used as a reference. (**L**-**M**) The semi-quantitative levels of iNOS and COX2. DEH treatment vs. GSK717 + DEH. (**N**) HCT116 cells were treated with DEH (40µM) in the presence or absence of GSK717 (20µM) for 24 h, and the expression levels of p62 was detected by western blotting. GAPDH was used as a reference. (**O**) The semi-quantitative levels of p62. DEH treatment vs. DEH + GSK717. Data are represented as mean ± SEM, *p < 0.05, **p < 0.01, ***p < 0.001, ****p < 0.0001

In LPS/IFNγ-stimulated RAW264.7 cells, different concentrations of DEH (5, 10 µM) or curcumin (5 µM) significantly inhibited levels of NOD2. Similarly, treatment with DEH (40 mg/kg) increased level of NOD2 in tumor tissues of BALB/c nude mice. Immunofluorescence results also showed a significant enhancement of NOD2 expression in tumor tissues after DEH (40 mg/kg) treatment. GSK717 is a known inhibitor of the NOD2 protein. To further explore whether the anti-inflammatory effects of DEH depend on NOD2, we observed whether the presence of GSK717 affects the inhibition of DEH on iNOS and COX2 production in RAW264.7 cells stimulated by LPS/IFNγ. As shown in Fig. 8K-M, GSK717 (5µM) weakened the inhibitory effects of DEH on iNOS and COX2 production induced by LPS/IFNγ in RAW264.7 cells. Simultaneously, the ability of DEH to promote increase in p62 protein levels in HCT116 cells was also partially attenuated by GSK717 (Fig. 8N-O). In summary, DEH can regulate inflammatory responses in RAW264.7 macrophages stimulated by LPS/IFNγ through NOD2. Additionally, it promotes autophagy in colorectal cancer by modulating NOD2.

## Discussion

The intestine is a unique environment in host defense, where inflammation plays a pivotal role in resolving pathogenic infections, maintaining normal intestinal function, or promoting tumorigenesis(Mantovani et al. 2008; Netea et al. 2017). According to the latest epidemiological reports, colorectal cancer ranks third in incidence and mortality among malignant tumors(Ciardiello et al. 2022). The time it takes for chronic inflammation in the intestines to progress to cancer is not a precise number. It may take anywhere from 10 to 30 years or even longer for chronic inflammation to lead to cancer. Research indicates that up to 42% of UC patients are found to have high-grade dysplasia, either already suffering from CRC or at risk of developing CRC in a short period of time. Therefore, controlling the chronic persistent inflammation of IBD and inhibiting the “inflammation-cancer transformation” are crucial steps in preventing and treating CRC. Although effective cancer screening measures have reduced the incidence and mortality of colorectal cancer, early-stage CRC can be cured through surgery. However, current treatments are not universally effective for every patient, and attempts to develop new drugs often fail due to incomplete understanding of the causes of IBD.

Pattern recognition receptors (PRRs) in the innate immune system play a crucial role. The nucleotide-binding oligomerization domain-containing protein (NOD)-like receptor (NLR) family is one of the main subfamilies of PRRs. They can influence autoimmunity, the progression of inflammatory diseases, and more(Trindade and Chen 2020). The nucleotide-binding oligomerization domain protein 2 (NOD2), also known as CARD15, is primarily expressed in monocytes, intestinal mucosal epithelium, Paneth cells, and dendritic cells. NOD2 contains tandem caspase recruitment and activation domains (CARDs), a nucleotide-binding oligomerization domain (NOD), and a carboxy-terminal leucine-rich repeat (LRR) domain(Abraham and Cho 2006). Upon activation by the conserved motif muramyl dipeptide (MDP), NOD2 undergoes self-oligomerization through its central NOD domain, inducing conformational changes in anti-NODs monomers and oligomerization via the NOD domain. This process facilitates the recruitment and activation of receptor-interacting protein 2 (RIP2), promoting autophagy responses or non-canonical NF-κB activation, leading to NF-κB translocation to the nucleus and promoting the transcriptional expression of various inflammatory mediators(Caruso et al. 2014). NOD2, as a key gene involved in inflammatory responses and regulating autophagy(Fritz et al. 2011; Mukherjee et al. 2019; Negroni et al. 2018), was the first susceptibility gene identified in association with inflammatory bowel disease(Branquinho et al. 2016; Goethel et al. 2019; Venkataraman and Rivas 2019). It is also considered a mediator of a range of human metabolic diseases including obesity, diabetes, non-alcoholic fatty liver disease, and metabolic syndrome(Zangara et al. 2021). Some scholars even believe that the gene-mediated nuclear autophagy leading to DNA damage can promote the occurrence of liver cancer(Zhou et al. 2021). Many studies consider NOD2 to be one of the potential risk factors that promote cancer occurrence. In this study, it was found that lower concentrations of DEH significantly inhibit inflammation in the inflammatory model established on RAW264.7 cells. Furthermore, DEH achieved its anti-inflammatory effects by suppressing the activation of NF-κB and MAPK signaling pathways. In vivo studies using UC animal models also demonstrated favorable anti-inflammatory effect. Subsequent exploration further revealed that DEH can inhibit the increased expression of NOD2 induced by LPS/IFNγ. This finding suggests that DEH may exert its anti-inflammatory effects through the suppression of NOD2 expression. In animal models in vivo, the intestinal mucosal mechanical barrier is primarily composed of intestinal epithelial cells and their tight junction (TJ) components. Among them, TJ represents the most crucial intercellular connection(Chen et al. 2021). Occludin is one of the main components of the intestinal mucosal mechanical barrier. By binding with ZO-1, it seals the intercellular gaps, forming an anti-permeability barrier to defend against pathogenic microbial invasion, which is crucial for maintaining the function of the intestinal mechanical barrier. The interaction between Occludin and ZO-1 proteins can regulate intracellular and extracellular signal transduction, affecting the integrity of tight junction (TJ) proteins, thereby modulating intestinal mucosal permeability. ZO-1 (Zonula Occludens-1) protein belongs to the membrane-associated guanylate kinase protein family and is involved in regulating intracellular material transport, maintaining epithelial polarity. It plays a role in cell proliferation and differentiation as well as in the migration of tumorigenic cells(Li et al. 2020a, b). In rat models of ulcerative colitis (UC), the expression of ZO-1 protein is significantly reduced. In vivo studies have found that DEH can effectively improve the dysregulation of TJ proteins in UC models. This demonstrates that DEH exhibits anti-inflammatory effects when used at low doses. Furthermore, this effect appears to be associated with the inhibition of NOD2 expression. After inhibiting NOD2 expression, the efficacy of DEH decreased, suggesting that low doses of DEH may primarily exert anti-inflammatory effects through the inhibition of NOD2.

Interestingly, subsequent studies investigated the anticancer effects of DEH on colorectal cancer cells, revealing that at high doses, DEH significantly inhibits the proliferation of HCT116 cells and promotes apoptosis of cancer cells. The metastasis of colorectal cancer cells is one of the primary reasons for high mortality rates in colorectal cancer. Epithelial-mesenchymal transition (EMT) is a major mechanism of tumor migration, enhancing the aggressiveness of colorectal cells following EMT. As a promoter of tumor invasion, loss of E-cadherin disrupts cell-cell adhesion(Nagy et al. 2022). In this experiment, it was found that DEH can effectively promote the expression of E-cadherin, reduce the expression of N-cadherin, thereby inhibiting tumor invasion and migration. Cyclin-dependent kinase 4 (CDK4) and cyclin-dependent kinase (CDK6) are key mediators for cell transition into the S phase. Constitutive activation of CDK4/6 represents a driving force in the onset of several cancer types(Goel et al. 2022). Researchers have found that CDK4/6 inhibitors not only suppress cell proliferation and trigger anti-tumor immune responses(Deng et al. 2018; Goel et al. 2017; Zhang et al. 2018), but also inhibit tumor growth through mechanisms including regulating mitotic kinase signaling, inducing senescent-like phenotypes(Wang et al. 2022), and enhancing the immunogenicity of cancer cells(Goel et al. 2018). p21 is a low molecular weight protein (21 kDa) transcribed from the CDKN1A gene. Initially identified as a cyclin-dependent kinase (CDK) regulator, it plays a crucial role in controlling cell cycle progression(Kuang et al. 2021; Wade Harper 1993). p21 binds to and inhibits cyclin-CDK1, CDK2, CDK4, and CDK6 complexes, thereby halting cell cycle progression at the G1 and S phases(Bertoli et al. 2013; Georgakilas et al. 2017). Increased expression of p21 leads to cell growth arrest at the G2 phase(Niculescu et al. 1998; Thoma et al. 2024). p21 can irreversibly halt the cell cycle in response to extensive DNA damage, serving as a protective mechanism against cancer development(Kumari and Jat 2021). Experimental results indicate that high doses of DEH can also suppress the expression of CDK4, CDK6, and p21, regulating the cell cycle, inducing cell cycle arrest in cancer cells, and promoting apoptosis in cancer cells.

Subsequently, this study found that DEH can significantly induce autophagy in colorectal cancer cells, leading to a significant increase in LC3B and p62 expression. Autophagy is a self-degradation mechanism widely present in all cells of the human body. Under normal physiological conditions, cells utilize autophagy to degrade and eliminate abnormal proteins, damaged organelles, and other substances. The breakdown products are then reused by the cell for its own life processes, maintaining cellular homeostasis. Extensive research has demonstrated the indispensable role of physiological autophagy in the initiation and progression of tumors(Duan et al. 2021; Xu et al. 2021). Most researchers believe that autophagy plays a “double-edged sword” role in the initiation and progression of tumors. In the early stages of tumor development, autophagy serves as a mechanism for quality control and survival promotion, clearing out risk factors such as genetic mutations and misfolded proteins, thereby preventing tumor formation and suppressing cancer progression. As tumors progress to later stages and face environmental stress, autophagy within tumor cells continues to operate as a mechanism that promotes survival. It serves as a robust support for tumor survival and growth by functioning as a degradation and recycling system(X. Li et al. 2020a, b). This has led many researchers to focus on autophagy, attempting to find new strategies to combat tumors by either activating or inhibiting autophagy. However, due to autophagy’s inherent survival-promoting characteristics, most scholars consider inhibiting autophagy as an adjunctive therapy to reduce drug resistance. When combined with radiation, chemotherapy, and other targeted therapies, this approach often achieves good results(Amaravadi et al. 2019). However, there is limited research on the strategy of activating autophagy to combat cancer, and it has only been applied in some studies of immunotherapy. Previous studies have shown that NOD2 plays a crucial role in the autophagy process. NOD2 interacts with autophagy-related proteins to target sites of pathogen invasion within cells. This interaction further leads to the formation of the ATG16L1-ATG12-ATG5 complex, promoting the conversion of LC3-I to LC3-II in the cytoplasm. An increase in LC3-II levels indicates an increase in autophagosome formation and cellular autophagic activity. Interestingly, this study found that a high dose of 50µM DEH significantly upregulates NOD2 expression, leading to the induction of autophagy. When NOD2 expression was blocked, the induction of autophagy by DEH was attenuated, suggesting that DEH-induced cell death in colorectal cancer cells at high concentrations may occur through the activation of NOD2 expression. Subsequently, in animal models, we also observed that DEH significantly inhibited the growth of subcutaneous colorectal cancer xenograft tumors. Furthermore, it activated the expression of LC3B and NOD2, demonstrating that DEH can promote autophagy by enhancing NOD2 expression both in vitro and in vivo.

As is well known, natural compounds and their derivatives extracted from traditional Chinese herbs are considered ideal alternatives to anticancer drugs due to their advantages such as low cost, strong efficacy, and minimal side effects. The ‘inflammation-to-cancer transformation’ in the intestines has long been a troubling issue. How to effectively control inflammation and suppress the onset of cancer remains a topic of research. Traditional Chinese medicine often plays a double-edged sword role in clinical use. In this experiment, we discovered that DEH, as an active component of nutmeg, can inhibit the production of inflammatory responses at low concentrations and has a significant anti-inflammatory effect in an in vivo UC model. At high concentrations, it can significantly induce apoptosis and autophagy in colorectal cancer cells. Furthermore, these phenomena are all associated with the regulation of NOD2 expression. During inflammation, DEH can act by inhibiting the excessive expression of NOD2. At high concentrations, it can induce autophagy by promoting the expression of NOD2.

## Conclusion

In summary, this study identifies DEH as a novel small-molecule compound with dual therapeutic effects against both colitis and CRC, demonstrating significant therapeutic potential in targeting the inflammation-cancer transition within colorectal pathologies. Mechanistically, DEH exerts bidirectional regulation of NOD2: at lower concentrations, it suppresses NOD2 expression to potently inhibit colitis-associated inflammation, whereas at relatively higher concentrations, it activates NOD2 to induce autophagy in CRC cells, thereby achieving anticancer efficacy. These findings provide novel insights into the concentration-dependent bidirectional regulation of target proteins and different disease by natural product.

## Electronic supplementary material

Below is the link to the electronic supplementary material.

Supplementary Material 1

Supplementary Material 2

## Data Availability

No datasets were generated or analysed during the current study.

## Abbreviations

IBD: Inflammatory bowel disease
CRC: colorectal cancer
CD: Crohn’s disease
UC: Ulcerative colitis
DSS: dextran sulfate sodium
MAPK: Mitogen-activated protein kinase
ERks: Extracellular regulated protein kinases
NF-κB: Nuclear factor kappa B
PPARs: Peroxisome-proliferator-activated receptors
RT-qPCR: Real-time quantitative polymerase chain reaction
IF: Immunofluorescence
IHC: Immunohistochemistry
