# Supplementary figures and images for: Dehydrodiisoeugenol targets NOD2 exerting dual effects against colitis and colorectal cancer: a double-edged sword
Source: Mol Med. 2025 Jun 5;31:221. doi: 10.1186/s10020-025-01193-7 (PMC12139060; doi:10.1186/s10020-025-01193-7)

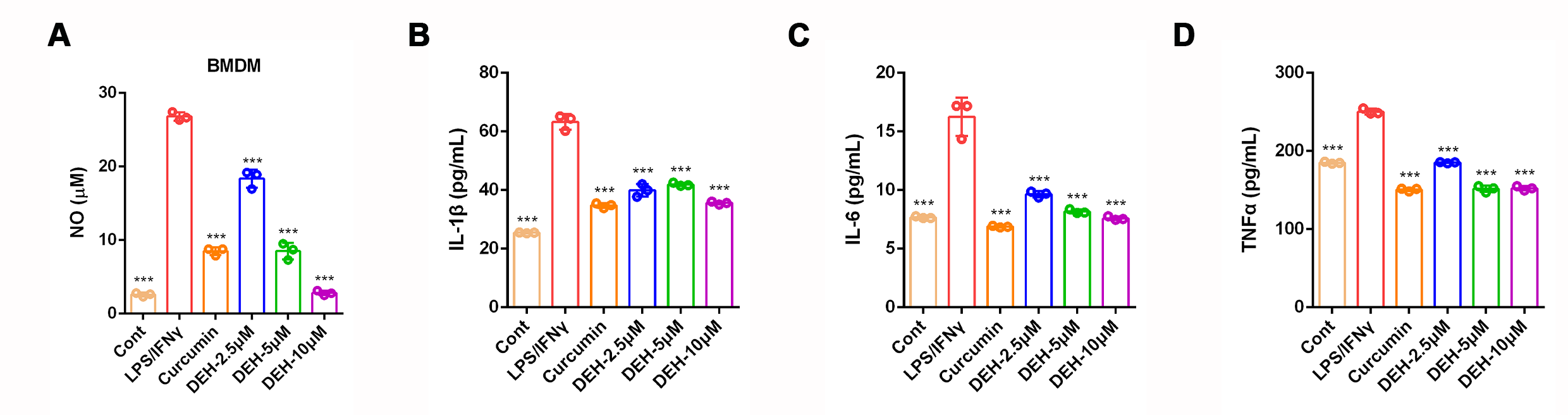

Supplement: Supplementary file 1 — Supplementary Material 1 [file 10020_2025_1193_MOESM1_ESM.tif]
