# Supplementary material for: Dehydrodiisoeugenol targets NOD2 exerting dual effects against colitis and colorectal cancer: a double-edged sword
Source: Mol Med. 2025 Jun 5;31:221. doi: 10.1186/s10020-025-01193-7 (PMC12139060; doi:10.1186/s10020-025-01193-7)
Supplement: Supplementary file 2 — Supplementary Material 2 [file 10020_2025_1193_MOESM2_ESM.docx]

Supplementary Fig. 1: DEH inhibits the NO production of BMDMs stimulated with LPS/IFNγ, as well as inhibits the inflammatory factors release of RAW264.7 stimulated with LPS/IFNγ. (A) The effect of DEH on NO production in LPS (0.5μg/ml)/IFNγ (10ng/ml) stimulated bone marrow derived macrophages (BMDMs). (B-D) RAW264.7 cells were pretreated with DEH (2.5, 5, 10μM), following stimulated with LPS (0.5μg/ml)/IFNγ (10ng/ml), and then the levels of IL-1β, IL-6 and TNFα in cellular supernatant were measured using ELISA assay.
